# A Low-Power Integrated Humidity CMOS Sensor by Printing-on-Chip Technology

**DOI:** 10.3390/s140509247

**Published:** 2014-05-23

**Authors:** Chang-Hung Lee, Wen-Yu Chuang, Melissa A. Cowan, Wen-Jung Wu, Chih-Ting Lin

**Affiliations:** 1 Graduate Institute of Electronics Engineering, National Taiwan University, Taipei 10617, Taiwan; E-Mails: d98943030@ntu.edu.tw (C.-H.L.); muki1428@gmail.com (W.-Y.C.); 2 Intel Lab, Hillsboro, OR 97124, USA; E-Mail: melissa.a.cowan@intel.com; 3 Department of Engineering Science and Ocean Engineering, National Taiwan University, Taipei 10617, Taiwan; E-Mail: wjwu@ntumems.net

**Keywords:** sensor integration, inkjet printing, gas sensor

## Abstract

A low-power, wide-dynamic-range integrated humidity sensing chip is implemented using a printable polymer sensing material with an on-chip pulse-width-modulation interface circuit. By using the inkjet printing technique, poly(3,4-ethylene-dioxythiophene)/polystyrene sulfonate that has humidity sensing features can be printed onto the top metal layer of a 0.35 μm CMOS IC. The developed printing-on-chip humidity sensor achieves a heterogeneous three dimensional sensor system-on-chip architecture. The humidity sensing of the implemented printing-on-chip sensor system is experimentally tested. The sensor shows a sensitivity of 0.98% to humidity in the atmosphere. The maximum dynamic range of the readout circuit is 9.8 MΩ, which can be further tuned by the frequency of input signal to fit the requirement of the resistance of printed sensor. The power consumption keeps only 154 μW. This printing-on-chip sensor provides a practical solution to fulfill an ultra-small integrated sensor for the applications in miniaturized sensing systems.

## 1. Introduction

In the domain of machine-to-machine (M2M) networks, sensing devices are fundamental for versatile context awareness applications, such as factory automation, home environment monitoring, and healthcare [1,2]. Among different kinds of sensing quantities, gas sensing, e.g., e-nose systems, are a big topic for applications in indoor-air-quality (IAQ) monitoring [3] and medical care [4]. A numbers of technologies have been exploited to develop novel gas sensors which can offer miniaturized and low-power consumption characteristics [5–9]. However, most gas sensor systems are limited by the dimensions of discrete sensor devices, or the power consumption is still too large to combine with batteries or self-sustaining power harvesters for long-term usage [10]. As a consequence, building a low-power, highly integrated sensor system is necessary to conquer these issues.

To achieve above features, gas-sensitive polymer materials are a good choice for the low-power requirements because polymer sensors can be operated without heaters to reduce the power consumption [11,12]. In addition, polymers could be easily blended with other organic/inorganic materials to alter the sensing target, which means the selectivity of sensors for specific applications can be improved [12]. On the other hand, polymer-based gas sensors are attractive because they can be fabricated by diverse methods, which provide high possibilities and flexibility in engineering [13,14]. Among all the fabrication methods for polymers, we focus on the inkjet printing technique because of its drop-on-demand, non-contact, no-mask features, and simple integration with other platforms [15,16]. These advantages of inkjet printing offer a fast-prototyping method to fabricate organic electronic devices of high complexity. However, the printed circuits still suffer from lower speed and higher process variation compare to standard CMOS ICs. Therefore, we have demonstrated a three-dimension stamping method and standard CMOS technology to take advantage of both the computational speed strengths of CMOS and the versatile sensing functions of polymer-based materials in previous work [17]. In a step further, in this work, the inkjet printing technique was used to achieve more accurate positioning to realize multi-printing in a small form factor.

In this paper, we propose a heterogeneous integration platform for gas-sensing, which could implement a small-form-factor and a low-power system with ease. Using the additive characteristics of the inkjet printing process, a pseudo-3D integrated circuit was prepared. Using this method, the circuit area could be vertically combined with the sensor device. A readout CMOS chip was specifically designed for inkjet printing integration. This simple pulse-width-modulation (PWM) readout architecture also has the potential for extending it to multi-sensor array topologies.

## 2. Experimental Section

### 2.1. Circuit Architecture

**Figure 1.**
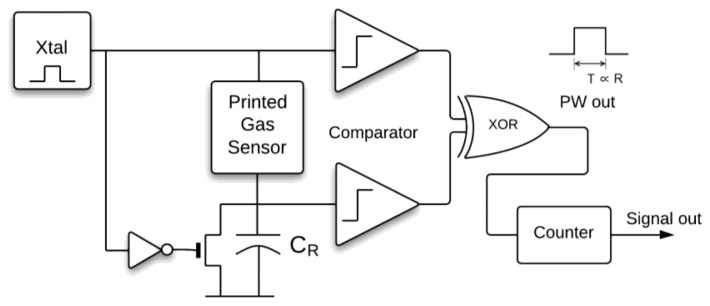
The block diagram of the proposed PWM sensor readout IC, the output pulse width (T) is proportional to the resistance of printed sensor (R).

The resistance of the printed sensor could respond to the gas concentration over a huge range (from 10^4^ to 10^6^ Ω). As a consequence, the interface circuit needs to adapt to the variation of the printing process. To overcome this issue, we choose a wide-dynamic-range and low-power readout circuit to implement the integrated readout circuits [18]. Compared to the voltage-divider architecture, this architecture can prevent the reference matching and minimize the distortion as output nears the boundary of the circuit dynamic range [19]. The block diagram shown in Figure 1 represents the configuration of the designed resistive-type sensor readout. The principles of operation and detailed design have been discussed in [15]. This semi-digitized PWM readout circuit linearly converts the resistance of the printed sensor to the output pulse width. The pulse width is then converted by a counter to a full-digitized output with the RS-232 protocol. The dynamic range of the readout circuit can be controlled by the reference capacitor (C_R_), the frequency, and the duty cycle of input signals. In this design, we set the input frequency as 32.768 kHz, which can be simply implemented by a crystal oscillator. The C_R_ is designed as 2 pF. With these conditions, the designed dynamic range is R_max_ = 9.8 MΩ; R_min_ ∼ 500 kΩ; the resolution is 1.5 ns/kΩ.

### 2.2. Fabrication of CMOS Chip

**Figure 2.**
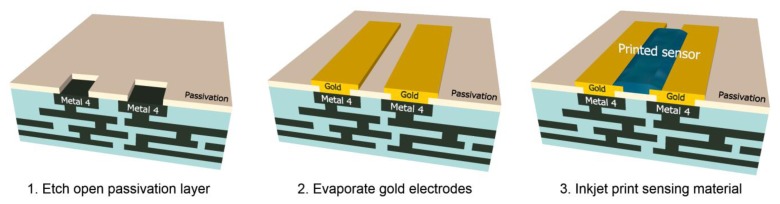
The 3D illustrations of on-chip post-process steps.

**Figure 3.**
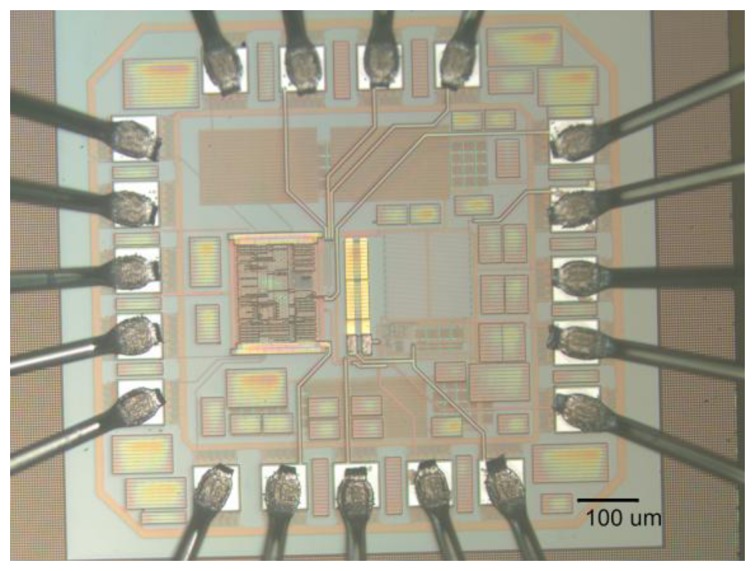
Microscopic image of the sensor IC printed sensor, the gold electrode pair in the center locates the printed area for sensor.

The sensor interface chip was designed and fabricated on a 0.35 μm CMOS Bio-MEMS platform, which includes a gold layer post-process step. The gold layer is vital for connecting the polymer sensor device because gold has good compatibility with conductive polymers [20]. After the fabrication of CMOS circuits, the chip was etched to open a contact window to the top metal layer (Metal 4). Then the gold layer was deposited to form an electrode pair according to the pattern layout. These gold electrodes could conduct the signal from the top sensor to the readout circuits. These post-process steps are shown in Figure 2, and the implemented chip photo is shown in Figure 3. The die area is 0.5 × 0.57 mm^2^, not including bounding pads. The dimension of each gold electrode pair was designed to 200 μm × 20 μm, with a 200 μm (W) × 10 μm (L) gap. This circuit also fulfilled low-power requirement, the simulated average power consumption is 135 μW, without full-digitized conversion block. The simulated full function power consumption is 400 μW.

### 2.3. Inkjet-Printed Sensor on CMOS Chip, Material and Fabrication

A gas sensing material, we chose poly(3,4-ethylenedioxythiophene)/polystyrene sulfonate (PEDOT:PSS), a widely used conductive polymer, as the backbone ingredient. PEDOT:PSS composite materials could sense many kinds of gases, such as water [21], NO_x_ [22], and CO [23]. The aqueous nature of PEDOT:PSS also is a good feature for the printing process. To demonstrate the discrimination of different sensing materials toward different gases, aluminum doped zinc oxide (AZO) nanoparticles were added into PEDOT:PSS to form a composite sensing material, which was verified in previous works [24,25]. Pristine PEDOT:PSS and blended PEDOT:PSS/AZO were printed onto the gold electrodes of CMOS chips by a self-made inkjet printing system. Specifically, the PEDOT:PSS concentration was 2.5 wt% and the concentration of nanoparticles was 0.0125 wt% for the inkjet-printed sensing material [24]. The smallest dot size of the inkjet printing system is 50 μm in radius. Therefore, the inkjet printing system can be used to print a straight line onto the chip to adequately cover the gap of the designed electrodes. After printing the sensing material, the chip is soft baked and wire-bonded for further tests.

### 2.4. Gas Sensing Test Flow

Each sensor was tested in a customized sealed chamber under different gas conditions. For CO_2_, NO and CO tests, these testing gases were pre-mixed with N_2_ in different concentrations. The mixed gases were flushed into the chamber for 10 min. After 10 min, the chamber was evacuated by vacuum pump and flushed with N_2_ for 10 min. This sequence was repeated several times to complete the sensitivity test. For the humidity sensing tests, in addition, the test gas was composed of N_2_ pumped through a buffer tank with heated water. Tuning the ratio of water vapor and dry N_2_ can control the humidity of the chamber. For the ethanol (EtOH) sensing test, a specific volume of ethanol was injected into the inlet by a microsyringe pump and flushed into the chamber with dry N_2_. All the sensor testing conditions were at a pressure of 1 atm under room temperature. The input signal and the supply voltage (3 V) were applied by a National Instrument data acquisition card (USB-6363). Moreover, the output signals were also captured by the same equipment. To monitor the humidity profile in real-time, a precise hygrometer, which has a built-in SHT11 (Sensirion) sensor chip, was installed in the chamber as a reference.

## 3. Results and Discussion

**Figure 4.**
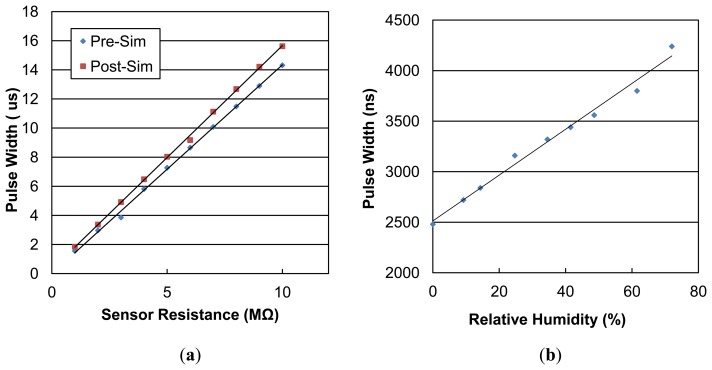
(**a**) Pre-simulation and post-simulation results of readout circuits. (**b**) The pristine PEDOT:PSS sensor response of relative humidity versus pulse width.

Figure 4a shows the simulated interface circuit results of the output pulse width *versus* resistance of the sensor. The discrepancy in the pre-simulation and post-simulation results is caused by the parasitic capacitances and resistances. This offset can be linearly calibrated after CMOS fabrication. For a 32 kHz, 50% duty cycle input signal, the maximum readout range is limited in 9.8 MΩ. The dynamic range can be adjusted to a higher value by simply altering the input duty cycle, or by decreasing the input frequency.

After the inkjet printing post-process, the sensor-integrated chip was tested under different kinds of gas conditions. The experimental data of the humidity-sensing test is shown in Figure 4b. With the different humidity levels injected into the chamber, the relative humidity (RH) increased from 0% to 72%, and the sensor demonstrated a linear response. The pulse width, which refers to the resistance of the sensor increased 70% under RH = 72%. From these results, we conclude that the PEDOT:PSS has a good sensitivity to humidity with about 0.98%/RH%. The wide-dynamic-range has covered the resistance shifting of the PEDOT:PSS sensor. The measured power consumption of the circuit system is around 154 μW, without a full-digitized conversion function. The measured total power consumption with full functionality is around 400 μW.

**Figure 5.**
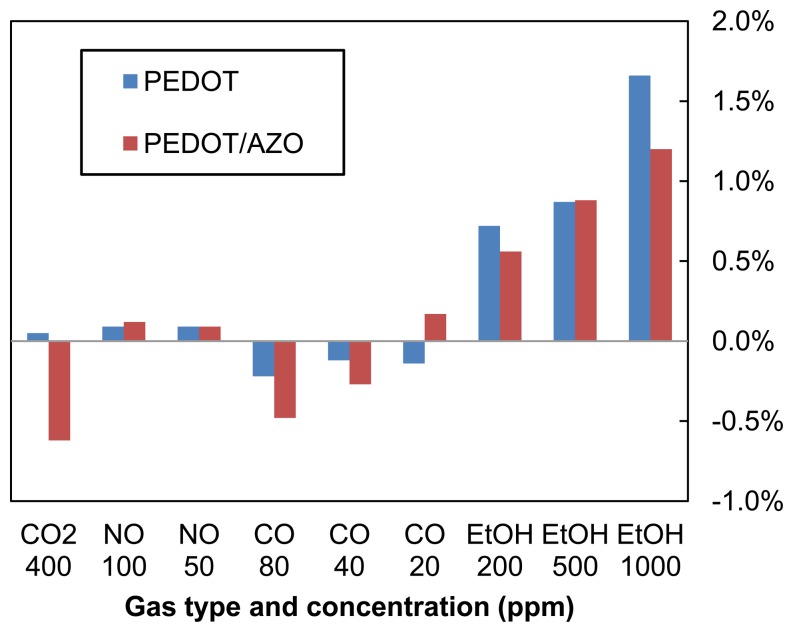
The sensitivity test in different kinds of gases with two types of sensors.

**Figure 6.**
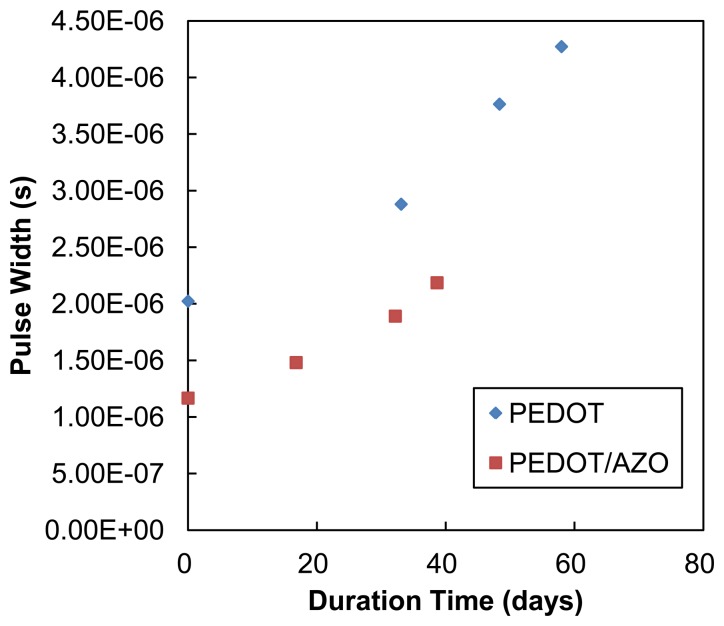
Stability test of two types of sensors.

To test the sensitivity and selectivity of PEDOT:PSS and PEDOT:PSS/AZO sensors against different gases, four kinds of gases including CO_2_, NO, CO, and EtOH were tested. The experimental results are shown in Figure 5. Both pristine and blended sensors show sensitivity to EtOH with over 1% variance at 1000 ppm. However, for CO_2_, CO, and NO, the sensors showed relatively weak responses at low concentrations. For the stability test, the two kinds of sensors were stored in an electronic dry box under 30% RH. The measured results are shown in Figure 6. The resistance of pristine PEDOT:PSS sensor has doubled after 60 days, and the resistance of the AZO/PEDOT:PSS blended sensor has doubled after 40 days. This degradation is caused by the moisture and oxygen, which would change the structure of the PEDOT:PSS polymer chains [26].

The specifications of the implemented chip and a comparison with the state-of-the art designs for similar purposes are summarized in Table 1 [27–29]. Compared with the referenced works, the proposed design demonstrated relatively low-power, room-temperature operation, and high versatility by printing different materials onto the sensing electrodes. Additionally, this design could be further improved by adding a selection circuit and a reference resistance to implement a dual-type readout, which can adapt to both resistive and capacitive sensing materials.

## 4. Conclusions

A prototype of printed humidity sensor-on-chip is presented. Implemented by an inkjet printing technique, polymer-based gas sensing materials were 3D stacked on the top of CMOS circuits. The CMOS chip is fabricated by the TSMC 0.35 μm BioMEMS 2P4M CMOS process, and the inkjet printing is accomplished using a self-made inkjet printer. With a low power consumption of 154 μW from a 3 V supply, the experimental results demonstrate that the sensors have good sensitivity for humidity and alcohol. The core chip area is only 0.5 × 0.57 mm^2^. The small-form-factor and low-power integrated sensor chip shows the potential to achieve multi-sensors on a single chip for M2M networks applications in IAQ monitoring or healthcare.

## Figures and Tables

**Table 1.** Specification Comparison.

	**2007 [27]**	**2009 [28]**	**2012 [29]**	**Proposed**
Technology	5 um	0.35 um	0.35 um	0.35 um
Supply Voltage	10	N/A	3.3	3
Power	15.5 mW	30 mW	1.9 mW	0.4 mW (154 μW only PW)
Gas sensor	SnO_2_	SnO_2_	Polycarbazole	Printable polymer
Method	Differential readout	Oscillator	impedance spectroscopy	Pulsewidth modulation
Operation Temperature	300 °C	400 °C	27 °C	27 °C
